# Second District Dental Society of New York

**Published:** 1872-05

**Authors:** 


### Second District Dental Society of New York.

The annual meeting of this Society was held in Brooklyn, on Tuesday, April 9th, 1872.

After some miscellaneous business had been transacted, the election of officers was held with the following result:

President, Dr. O. E. Hill, of Brooklyn; Vice President, Dr. T. C. Royce, of Middletown; Treasurer, Dr. H. G. Mirick, of Brooklyn; Rec. Sec., Dr. M. E. Elmendorf, of Brooklyn; Cor. Sec., Dr. F. W. Dolbeare, of Brooklyn; Librarian, Dr. Geo. A. Mills, of Brooklyn.

Delegates to the State Society—Dr. W. B. Hurd, Dr. W. S. Elliott.

In the course of the proceedings, a call was made upon the committee appointed by the Convention of Dentists, in December, 1871, for information in regard to the rubber suits. This call or request being approved by the Society, Dr. Hill and Dr. Northrup, who were present, gave a detailed statement of the entire proceedings of the committee since their appointment, of the difficulties they had encountered, of the presentation and argument of the case in the Supreme Court, and of the hopes of success which the committee entertained.

The annual dinner was given in the evening, at which many distinguished visitors were present, and among them Rev. H. Moore, of Cincinnati.

Upon reconvening, the President elect was formally inducted into the chair and the remaining business of the meeting transacted.

The following resolution was then offered by Dr. Marvin, and unanimously adopted, with the direction that it be offered to several dental journals for publication,

*Resolved*, That the thanks of this Society are hereby tendered to the committee appointed by the Convention of Dentists, held in New York City in December last, to defend the interests of the profession in the matter of the Rubber Patents; and also to the counsel employed by said committee for the very thorough and able manner in which those interests were vindicated before the Supreme Court of the United States; and let the result be what it may, we hereby record our approval of their action, our thanks for their unwearied efforts, and our sympathy with them in all the difficulties and under all the adverse circumstances which they have met in the execution of their trust.

The meeting then adjourned.

M. E. Elmendorf, Sec'y.

---

At a meeting of the Brooklyn Dental Society, Monday evening, April 15, the following action was taken:

*Resolved*, That this Society affirm and endorse the resolution of thanks and approbation passed at the annual meeting of the Second District Society, April 9, as to the action of the committee on the Patent Suits and their counsel.

Jno. C. Wyman, Sec'y.

